# Cryogel scaffolds for localised delivery of lipopolysaccharide in organotypic spinal cord slice cultures: A novel *ex vivo* model of neuroinflammation

**DOI:** 10.1016/j.mtbio.2025.102211

**Published:** 2025-08-19

**Authors:** Ciara M. Walsh, Sophie Hill, Ben Newland, Dearbhaile Dooley

**Affiliations:** a School of Medicine, Health Sciences Centre, University College Dublin, Belfield, Dublin 4, Ireland; b UCD Conway Institute of Biomolecular & Biomedical Research, University College Dublin, Belfield, Dublin 4, Ireland; c School of Pharmacy and Pharmaceutical Sciences, Cardiff University, Redwood Building, King Edward VII Avenue, Cardiff, UK; d Leibniz-Institut für Polymerforschung Dresden e.V., 01069, Dresden, Germany

## Abstract

Spinal cord injury (SCI) is a devastating condition for which no curative therapy is currently available. The pathology of SCI is underscored by an inflammatory lesion at the site of injury that exacerbates damage and impedes recovery. Immunomodulation is a promising strategy for SCI repair and thus there is enhanced focus on identifying and testing novel immunotherapeutics. Efficient preclinical models are required for screening new therapies, and *ex vivo* models can reduce the overall cost and animal numbers required for this process. Organotypic spinal cord slices offer a promising *ex vivo* platform for modelling spinal cord pathologies as they retain the *in vivo* tissue architecture with the benefit of a controlled culture environment. Neuroinflammation can be induced in organotypic spinal cord slices by adding inflammatory agents to the culture system, however this results in global inflammation and lacks the heterogeneity of a focal lesion surrounded by spared tissue that is observed *in vivo*. To improve this model, we have applied previously characterised macroporous cryogels for localised delivery of lipopolysaccharide (LPS) in organotypic spinal cord slices. Placement of LPS-loaded cryogels adjacent to spinal cord slices increases the expression of proinflammatory CD86 in Iba-1^+^ microglia/macrophages and decreases the expression of myelin basic protein at the lesion site. These effects are not observed distal to the cryogel, indicating the formation of a focal inflammatory lesion. These effects can be reversed through treatment with the immunomodulatory cytokine interleukin(IL)-13. This novel model of neuroinflammation provides an innovative platform for screening potential immunotherapeutics and improving the efficiency of future preclinical SCI studies.

## Graphical abstract

**Figure:**
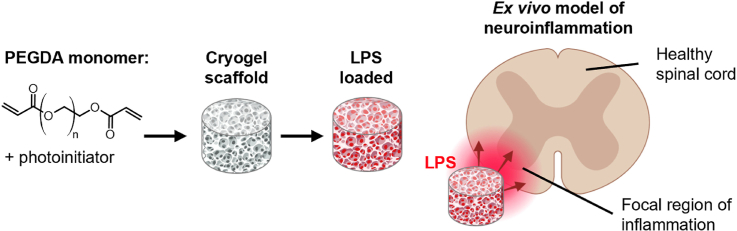

## 1 Introduction

Traumatic spinal cord injury (SCI) is a devastating condition causing sensory, motor and autonomic deficits in patients and has a severe impact worldwide [1]. SCI is characterised by a primary injury that occurs upon impact and leads to death of neural and glial cells at the site of damage [2]. This triggers a sequence of degenerative biological events that is collectively termed the secondary injury and can persist for months after the initial impact [2,3].

One of the major events of the secondary injury phase is the inflammatory cascade which mainly consists of local microglia and infiltrating peripheral macrophages [4]. Inflammation is crucial for recovery in SCI as microglia and macrophages function in phagocytosing dead cells and priming the injury microenvironment for regeneration [[5], [6], [7], [8]]. However, persistent inflammation can worsen recovery due to excessive secretion of proinflammatory cytokines that can trigger cell death and exacerbate the injury [7]. Modulation of the inflammatory response towards a more proregenerative state through the administration of immunomodulatory agents has proven to be a promising treatment strategy for SCI [9,10]. These immunomodulatory agents are tested in preclinical SCI studies using animal models [3]. While rodent SCI models provide a good platform for preclinical testing, no curative therapy has yet been clinically developed and thus further research into potential therapeutics is urgently needed.

Although rodent SCI models faithfully recapitulate many aspects of human SCI pathophysiology, they are not suitable for high or moderate throughput screening of candidate therapeutics. Therefore, an *in vitro* or *ex vivo* model of neuroinflammation may provide a more useful tool for screening immunomodulatory agents prior to full scale *in vivo* preclinical studies. Common *in vitro* models include cell lines, primary cells or induced pluripotent stem cell (iPSC)-derived cells which are treated with inflammatory agents in the culture media to induce inflammation [[11], [12], [13], [14]]. *In vitro* models provide a cost-effective platform for genetic manipulation, enabling mechanistic studies in a controlled environment [15]. However, the inflammatory cascade in SCI is a complex and multicellular phenomenon that cannot be accurately recapitulated using a single cell type model. Paracrine signalling and tissue-specific factors mean that neuroinflammation is best studied in a multicellular system that resembles physiological context. Much progress has been made in the use of 3D organoids as models of central nervous system (CNS) trauma [16]. For example, Van Breedam et al. subjected human iPSC-derived neurospheroids to 6 h of oxygen-glucose deprivation as a model of ischemic stroke and found that neural viability was significantly increased [17]. Daviaud et al. developed cerebral organoids from iPSCs of multiple sclerosis (MS) patients and were able to identify potential targets for therapeutic strategies [18]. However, various limitations persist in the organoid field including lack of standardised protocols, variation in organoid cytoarchitecture and inability to fully recapitulate the spinal cord microenvironment [16]. Most current protocols generate organoids that lack functional microglia which is a major barrier to their application in neuroinflammation research.

Organotypic spinal cord slices offer a multicellular *ex vivo* platform that overcomes the drawbacks associated with cell and organoid cultures. Organotypic slices fully recapitulate *in vivo* tissue architecture by retaining cellular organisation and interactions [[19], [20], [21], [22], [23]]. Critically, organotypic slices retain the endogenous microglia population which remain responsive to inflammatory stimuli [24]. Methods for inducing inflammation in organotypic slices from brain or spinal cord have traditionally focused on adding inflammatory agents, such as lipopolysaccharide (LPS), interferon gamma (IFN-γ) or tumour necrosis factor alpha (TNF-α), directly into the culture media [[24], [25], [26], [27], [28]]. However, this induces global inflammation throughout the entire slice which is not fully representative of an inflammatory SCI lesion whereby the lesion is surrounded by spared uninjured tissue. Therefore, a method of inducing a focal inflammatory lesion in an organotypic spinal cord slice would offer a novel *ex vivo* platform for modelling neuroinflammation in the context of SCI.

Previous work by Eigel et al. applied lysophosphatidylcholine (LPC)-loaded cryogel scaffolds to induce a focal demyelinated lesion in mouse brain and spinal cord slices as an *ex vivo* model for MS research [29]. There was minimal release of LPC from the scaffolds into the underlying media, allowing for focal delivery into the tissue slice and subsequent demyelination up to a depth of 394 μm ± 82 μm from the scaffold [29]. We therefore propose a similar approach whereby synthetic cryogels are loaded with LPS and placed adjacent to mouse organotypic spinal cord slices to induce a focal inflammatory lesion surrounded by spared non-inflamed tissue. Cryogels, or cryogelated hydrogels, are synthesised under freezing conditions to create a macroporous structure. This sponge-like strut and pore structure retains the soft compressible nature of standard hydrogels, but gives improved mechanical stability [30,31]. This is a key feature for their use in *ex vivo* models, where a material should be placed next to delicate tissue, yet be easily handled with forceps [29,32]. Applications of cryogels have included drug delivery [[33], [34], [35]], cell delivery [36,37], nerve regeneration [38,39] and focal manipulation of tissue in culture [29,32]. The cryogels applied in this study were synthesised from poly(ethylene glycol) diacrylate via 3D printed template/moulds to offer a reproducible and biocompatible platform from which to deliver LPS to the spinal cord.

To establish a novel *ex vivo* model of neuroinflammation using these cryogels, we first optimised the dosage of LPS required to induce inflammation in mouse organotypic spinal cord slices. We then loaded the previously characterised synthetic cryogels with this optimal dose of LPS before applying them to organotypic cultures to establish our novel *ex vivo* model of neuroinflammation. The inflammatory lesion was confirmed based on elevated expression of the proinflammatory marker CD86 in Iba-1^+^ microglia/macrophages adjacent to the cryogel scaffold. Treatment with the immunomodulatory cytokine interleukin(IL)-13, a known driver of an alternatively activated immune state [[40], [41], [42]], reduced inflammation at the lesion site. We therefore propose that this cryogel-mediated *ex vivo* model of neuroinflammation provides a novel platform for screening potential immunotherapeutics and enhancing the efficiency of preclinical SCI studies.

## 2 Materials & methods

### 2.1 Cryogel synthesis

Cryogels were synthesised in 3D printed moulds produced from PlasClear v2 resin (Asiga) using an Asiga Max-27 (Asiga). The moulds were designed using Autodesk Inventor software and printed in two parts (Fig. S1), to allow easy removal of the cryogels from the mould. The cylindrical moulds were 500 μm in diameter and 500 μm in depth. The parts were washed three times in isopropanol and post-cured with UV light (DR-301C, Asiga) for 30 min. The mould parts were plasma treated (PDC-003-CE, Harrick plasma) for 2 min prior to filling with the monomer precursor solution to prevent any adhesion of the cryogels to the template.

A monomer solution containing poly(ethylene glycol) diacrylate (PEGDA) (M_w_ 700) (Merck) at 10 % *w/v* was prepared in deionised water, to which the photoinitiator 2-hydroxy-2-methylpropiophenone (Merck) was added at a 1:35 M ratio to PEGDA. This solution was added to the mould via the filling wells to achieve equal filling of each cylindrical mould. The filled mould was then kept at −20 °C for 1 h, before being illuminated with UV light (395 nm, 2 × 15W bulbs (Kohler-Technik)), for 2 min to crosslink the monomers to a polymer network. The cryogels were removed from the template and added to 20 mL ethanol, which was replaced three times, to ensure no unreacted monomers or photoinitiator remained. The ethanol was then removed and the cryogels dried under vacuum overnight before use. For fluorescently labelled cryogels used in characterisation, iFluor **®** 647 Maleimide was added to the monomer solution (0.05 % v/v from a 10 mg/mL stock), and the rest of the synthesis/purification was carried out as above.

### 2.2 Cryogel characterisation

Cryogels were visualised in their dry form via scanning electron microscopy (SEM), by adding the filled (but dried) template, or the cryogels alone to an SEM stub with an adhesive carbon film. Samples were sputter coated with gold and imaged with a Tescan Vega SEM at 5 kV. Analysis of hydrated cryogels was performed with iFluor labelled cryogels on a Leica SP5 laser scanning confocal microscope using a HeNe laser for excitation at 633 nm. Eight cryogel samples were hydrated in PBS and imaged using a 20× air objective lens with a 20 μm Z-stack (interval distance of 3 μm) to obtain a projected average image representing a section through the base of the cryogel structure. ImageJ software (NIH) was used to measure cryogel diameter.

### 2.3 LPS loading and release assay

Dried cryogels were placed into Eppendorf tubes (LoBind, 0.5 mL, Eppendorf™ 0030108116), to which 200 μL of Alexa Fluor™ 488 labelled LPS (Invitrogen™, derived from *Escherichia coli* Serotype 055:B5, L23351) was added (25 μg/mL in PBS). The cryogels were loaded at room temperature for 10 min, before removal of the supernatant with a gel loading capillary pipette tip (Labcon Eclipse™, 16663892) to afford LPS loaded cryogels in their hydrated state. Subsequently, 200 μL of fresh PBS was added to initiate the release experiments, with removal and replenishment of the release medium at 15m, 1h, 2h, 4h and 24h. Release experiments were conducted at 37 °C. The global amount of LPS released into the media was quantified by measuring fluorescence using a TECAN Infinite® 200 Pro Series plate reader, with excitation and emission maxima of 495 nm and 530 nm, respectively. The fluorescence intensity was compared against a standard calibration curve to determine the corresponding LPS concentrations. At specific time points, the cryogels were placed into a 96 well plate and subjected to fluorescence imaging on the EVOS M700 imaging system (Invitrogen, 10x lens) utilising the GFP and brightfield channels.

### 2.4 Organotypic spinal cord slice isolation and culture

All housing and surgical procedures in this study were approved by the Animal Research Ethics Committee at University College Dublin and the Health Products Regulatory Authority of Ireland in accordance with the European Union Directive 2010/63/EU and S.I No. 543 of 2012. Postnatal day (P) 5 C57BL/6 mouse pups were humanely killed by decapitation and the spinal cord was immediately isolated. Briefly, skin and visible muscle was removed from the dorsum and the entire posterior column was removed and transferred to a fresh petri dish. The dorsal portion of the spinal column was removed under a stereomicroscope using spring scissors to expose the spinal cord, which was then gently removed using a fine spatula and transferred to a fresh petri dish with ice-cold Hank's balanced salt solution (HBSS, H8264, Merck) supplemented with 6 mg/mL glucose (25-037-CI, Corning). The meninges were gently dissected away using fine forceps and 350 μm transverse slices were cut using a McIlwain tissue chopper. Remaining steps were carried out in a biosafety cabinet under aseptic conditions. Whole, intact thoracic slices were transferred to 0.4 μm PTFE inserts (PICMORG50, Merck) that had been incubated in culture medium for at least 30 min. Residual liquid was removed from around the slice using a Pasteur pipette. Inserts were placed in a 6-well plate with 1 mL slice culture media below the insert (50 % minimum essential media [M4655, Merck], 25 % HBSS, 25 % horse serum [26050-070, Gibco], 20 mM HEPES [H0887, Sigma Aldrich], 6 mg/mL glucose, 1 % penicillin/streptomycin [15070-063, Gibco]) and cultured at 37 °C/5 % CO_2_ using the air liquid interface culture method. A full media change was performed after 24 h and every 2–3 days thereafter.

### 2.5 Slice treatment

All treatments were added at 6 days in culture. For global inflammation of spinal cord slice cultures, LPS was added directly to the culture media at 100, 500 or 1000 ng/mL. For cryogel-mediated LPS treatment, LPS was loaded into sterile cryogels by incubating cryogels in a 500 ng/mL LPS solution in sterile culture media for 10 min at room temperature. One cryogel was placed in direct contact with each spinal cord slice. Control cryogels were loaded with culture media. Treatments were left in place for 24 h. After 24 h, culture media was harvested and stored at −80 °C until analysis of LPS release.

### 2.6 Analysis of LPS release into culture medium

To confirm that LPS release from the loaded cryogels was limited to the adjacent tissue, and to ensure that LPS was not released into the underlying culture medium, the endotoxin content of the underlying culture medium was measured using the Pierce™ Chromogenic Endotoxin Quant Kit (A39552, Thermo Fisher). Following 24 h of organotypic spinal cord slice culture, 3 samples of culture medium per group were collected. The groups were as follows: *Control [media]* – culture medium only; *Control [LPS]* – culture medium with 500 ng/mL LPS; *Cryogel [media]* – culture medium from beneath media-loaded cryogels; *Cryogel [LPS]* – culture medium from beneath LPS-loaded cryogels. Endotoxin quantification was performed as per manufacturer's instructions.

### 2.7 Immunohistochemical staining and analysis

At 24 h post-treatment, slices were briefly washed in 1X PBS and then fixed for 1 h with 4 % paraformaldehyde (PFA) by placing 1 mL 4 % PFA below the insert and gently adding 1 mL 4 % PFA on top of the slices. Slices were then washed 3 times with 1X PBS and the mesh PTFE membrane containing the slices was cut from the plastic insert and transferred to a 24-well plate for staining. Slices were blocked and permeablised for 90 min with 5 % protein block (ab64226, Abcam) and 0.1 % Triton-X (306324N, BDH Laboratory Supplies Ltd) in 1X PBS. Primary antibodies (Table 1) were diluted in 1 % protein block and 0.05 % Triton-X in 1X PBS and incubated overnight at 4 °C. Slices were washed 3 times in 1X PBS and incubated in secondary antibodies (Table 1) for 2 h at room temperature. Slices were washed 3 times and counterstained with 300 nM Hoescht 33342 for 30 min at room temperature, followed by 3 washes with 1X PBS and 1 with distilled water. Slices were then inverted and mounted onto 35 mm glass bottomed dishes (75856-746, VWR) with Fluoromount mounting medium (F4680, Sigma Aldrich).

**Table 1:** Antibodies used for immunofluorescence.

Target	Host	Code	Supplier	Dilution
βIII-tubulin	Mouse	Ab7751	Abcam	1:1000
CD86	Rat	14-0862-82	Thermo Fisher	1:200
GFAP	Mouse	G3893	Sigma Aldrich	1:500
Iba-1	Rabbit	019–19741	Wako	1:300
MBP	Rat	MAB386	Merck	1:200
Anti-mouse 488	Goat	A32723	Thermo Fisher	1:300
Anti-rabbit 488	Goat	A11008	Thermo Fisher	1:300
Anti-rabbit 647	Goat	A32733	Thermo Fisher	1:300
Anti-rat 488	Goat	A11006	Thermo Fisher	1:300
Anti-rat 594	Goat	A11007	Thermo Fisher	1:300

For analysis of LPS-induced inflammation throughout the entire slice, one Z-stack was taken per spinal cord slice with a 5 μm step size from the top to the bottom of the slice to capture all emitted fluorescence using an Olympus FV3000 confocal microscope equipped with 405, 488, and 594 nm lasers and a 10× objective lens (Numerical Aperture = 0.4). Integrated fluorescent density of Iba-1, CD86 and GFAP were measured from a maximum projection within the slice ROI using ImageJ and non-specific fluorescence was excluded based on negative controls. For cryogel-treated slices, the lesion was identified based on the highest density of Iba-1 staining adjacent to where the cryogel was placed. One image within this region and one image directly opposite this site (“Distal” to the lesion) were captured using (i) a 10× objective lens and a zoom of x3.0 for Iba-1, CD86 and GFAP, or (ii) 20× objective for MBP and βIII-tubulin. Iba-1 and CD86 analysis was performed using CellProfiler [43] to identify Iba-1^+^ masks and measure the intensity of both markers within these regions. GFAP, MBP and βIII-tubulin analyses were performed using ImageJ by finding the average integrated fluorescent density of each marker within 3 randomly selected 100 × 100 μm regions adjacent to the edge of the slice. Analysis of βIII-tubulin axon counts and length was performed using the NeuronJ plug-in in ImageJ.

### 2.8 Statistical analysis

All statistical analyses were performed using GraphPad Prism 8.0 software. Data were tested for normality using the Shapiro-Wilk test. Details of statistical tests are given in the figure legends. Differences were considered statistically significant when p < 0.05. Data are presented as mean ± SEM.

## 3 Results

### 3.1 Induction of global inflammation in organotypic spinal cord slices with 500 ng/mL LPS

**Fig. 1:**
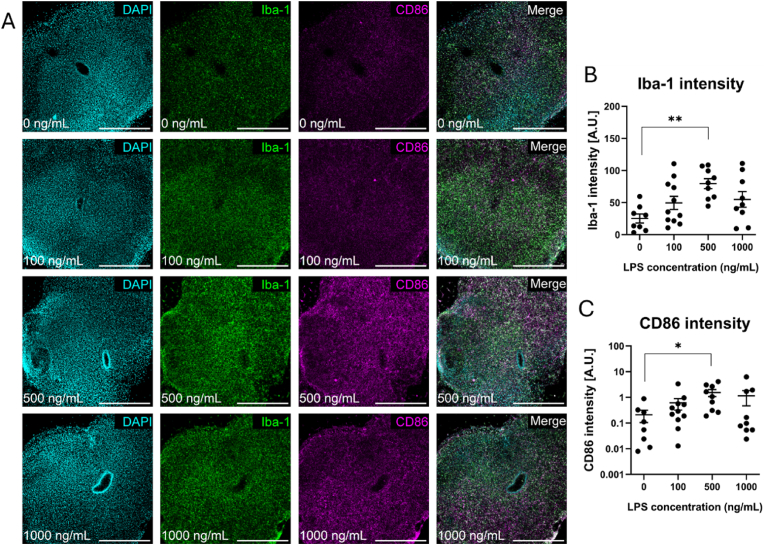
500 ng/mL LPS induces inflammation in organotypic spinal cord slices. (A) Representative photomicrographs of organotypic spinal cord slices treated with 0, 100, 500 or 1000 ng/mL LPS showing DAPI (cyan), Iba-1 (green) and CD86 (magenta). Intensity analysis of (B) Iba-1 and (C) CD86 reveal that 500 ng/mL induces significant increase in the intensity of both markers. Scale bars represent 500 μm. Data represent mean ± SEM of n = 9-12 slices from at least 3 mouse pups per condition. Analysis by one-way ANOVA with Tukey's multiple comparison's test, ∗p < 0.05, ∗∗p < 0.01. (For interpretation of the references to colour in this figure legend, the reader is referred to the Web version of this article.)

LPS ranging from 0 to 1000 ng/mL was added directly to the culture medium below the PTFE insert to confirm that LPS can induce inflammation in mouse organotypic spinal cord slices. Fig. 1 demonstrates that 500 ng/mL LPS induced an increase in the intensity of Iba-1 and CD86 in Iba-1^+^ microglia/macrophages, indicating that LPS can induce inflammation *ex vivo*. We did not observe any change in GFAP intensity following global LPS treatment, suggesting that LPS does not affect GFAP immunoreactivity in this model (Fig. S2).

### 3.2 Synthesis of cylindrical cryogels for localised LPS delivery

**Fig. 2:**
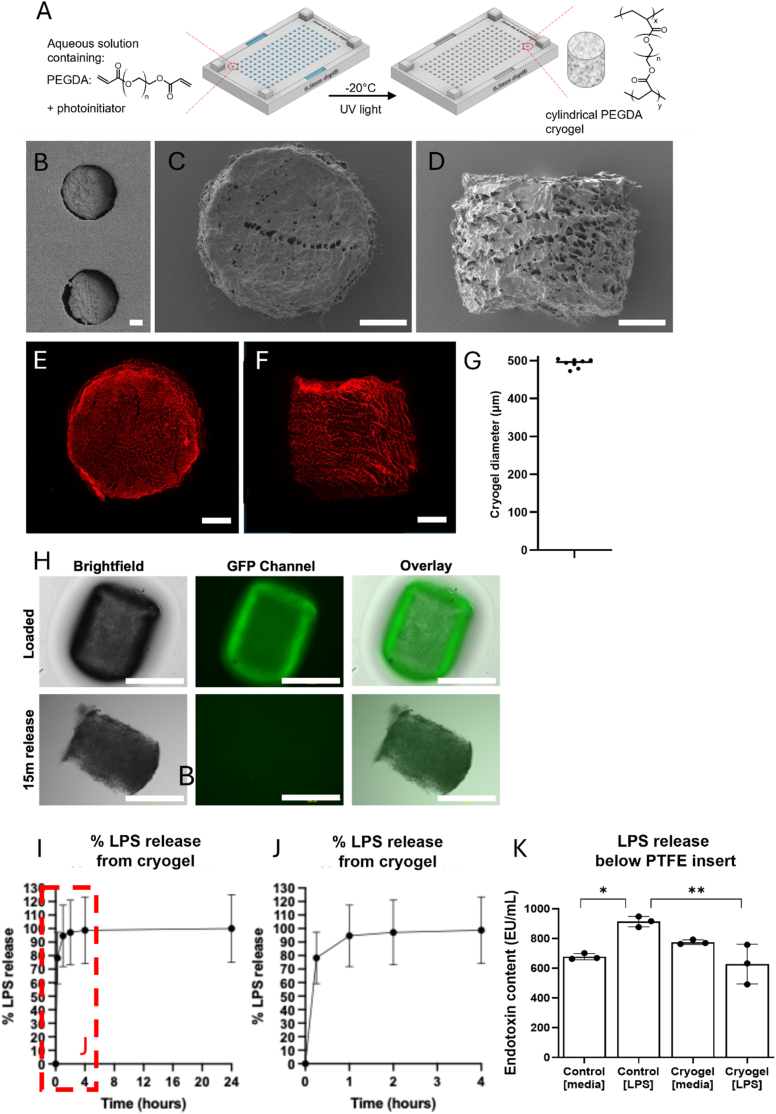
Synthesis and characterisation of cylindrical cryogels. (A) Schematic depiction of the cryogel synthesis process whereby the monomer precursor solution is added to the mould via the filling wells at the side, then frozen at −20 °C before being crosslinked via UV light. SEM images of (B) the cryogels whilst still in the mould, (C) the top of a cryogel cylinder, and (D) the side view. Confocal images of the hydrated cryogels (D) from above, and (E) from the side, with (F) subsequent analysis of the diameter of eight cryogels. All scale bars represent 100 μm. (H) Fluorescence images of cryogels immediately after loading and following 15 min of release of Alexa Fluor™ 488-labelled LPS. Scale bars represent 500 μm. (I) Release profile demonstrating the % release of Alexa Fluor™ 488-labelled LPS from the cryogels over 24 h, n = 3. Red dotted lines highlight rapid release within the initial 4 h shown in more detail in (J). (K) Analysis of endotoxin content of culture supernatant from untreated slices (Control [media]), LPS-treated slices (Control [LPS]), cryogel-treated slices (Cryogel [media], no LPS; Cryogel [LPS], LPS-loaded cryogel) shows that LPS-loaded cryogels do not release LPS into the culture supernatant, n = 3 samples/group. Analysis by one-way ANOVA with Tukey's multiple comparison's test, ∗p < 0.05, ∗∗p < 0.01. (For interpretation of the references to colour in this figure legend, the reader is referred to the Web version of this article.)

**Fig. 3:**
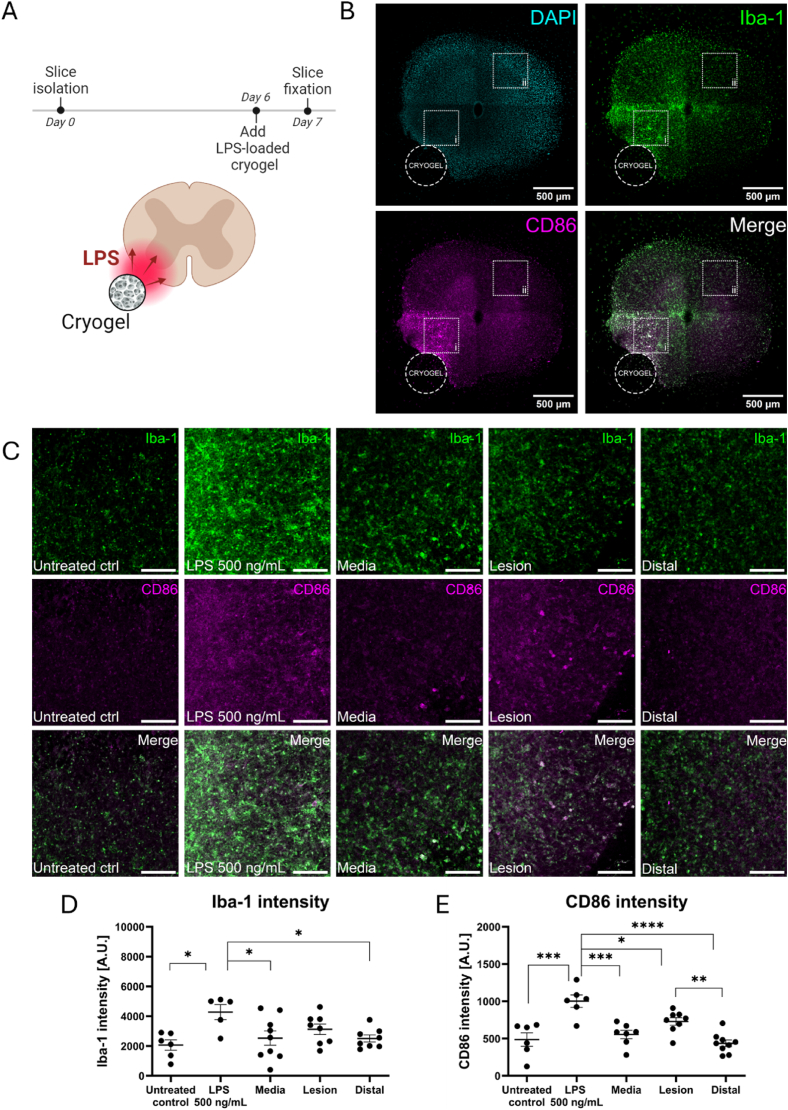
Cryogel-mediated delivery of LPS significantly increases CD86 expression in organotypic spinal cord slices. (A) Schematic of cryogel-mediated delivery of LPS to an organotypic spinal cord slice with the corresponding treatment timeline. (B) Overview of an organotypic slice following cryogel treatment showing DAPI (cyan), Iba-1 (green) and CD86 (magenta). Cryogel placement is shown as a dotted circle, and white squares outline the regions used for (i) lesion and (ii) distal analysis in (D–F). Scale bar represents 500 μm. (C) Representative photomicrographs of organotypic spinal cord slices from the following treatment groups: Untreated controls, LPS 500 ng/mL (LPS added directly to media), Media (treatment with media-loaded cryogel), Lesion (lesion region following treatment with LPS-loaded cryogel), Distal (distal region following treatment with LPS-loaded cryogel). Scale bars represent 100 μm. Quantification of (D) Iba-1 and (E) CD86 intensity was performed using CellProfiler. Data represent mean ± SEM of n = 6-9 slices from 3 mouse pups per condition. Analysis by one-way ANOVA with Tukey's multiple comparison's test, ∗p < 0.05, ∗∗p < 0.01, ∗∗∗p < 0.001, ∗∗∗∗p < 0.0001, ns p > 0.05. (For interpretation of the references to colour in this figure legend, the reader is referred to the Web version of this article.)

We then theorised that PEGDA cryogels could be used for localised delivery of LPS to organotypic spinal cord slices. PEGDA cryogels were successfully created in a 3D printed mould as outlined in Fig. 2A. Cryogels had a porous structure as shown by SEM (Fig. 2B–D) and confocal microscopy (Fig. 2E and F) and had an average diameter of 492 ± 11 μm (Fig. 2G). This tight control over the size allows delivery of repeatable quantities of liquid in subsequent experiments. Fluorescently labelled LPS was loaded into the cryogels and imaged both immediately after loading and 15 min after loading to demonstrate the successful loading and release of LPS from the cryogels (Fig. 2H). Analysis of LPS release from the cryogels over 24 h shows a rapid release within the initial hours after loading, suggesting limited retention of LPS by the cryogel scaffold which is supported by the fluorescence imaging data (Fig. 2H and I). LPS was rapidly released from the cryogel over 24 h with virtually 100 % released within the initial 4 h (Fig. 2I and J). This demonstrates the lack of electrostatic interaction between the LPS and the cryogel. A cryogel size of 0.5 μm diameter and 0.5 μm height holds a total volume of 0.098 μL. With a loading solution of 500 ng/mL used in subsequent biological experiments, this equates to a delivery of 0.049 ng LPS per cryogel directly to the tissue. We then measured the endotoxin content of culture medium from experiments outlined in Fig. 3 to assess if LPS was released from the cryogels into the underlying culture medium. Endotoxin content of medium from LPS-loaded cryogel stimulated cultures did not differ from untreated LPS-free culture medium, and was significantly lower than culture medium containing 500 ng/mL LPS (Fig. 2K). This suggests that LPS released from the cryogel is not released into the underlying culture medium, thereby preventing inadvertant global stimulation.

### 3.3 Cryogel-mediated delivery of LPS induces a focal inflammatory lesion in organotypic spinal cord slices

To create a novel *ex vivo* model of neuroinflammation, cryogel scaffolds were loaded with LPS and placed adjacent to mouse organotypic spinal cord slices for 24 h (Fig. 3A). Slices were then immunofluorescently stained for Iba-1 and CD86 to visualise the formation of an inflammatory lesion in the region adjacent to the cryogel scaffold (Fig. 3B). Although there was no difference in Iba-1 intensity between the ‘lesion’ and the ‘distal’ tissue, there was a significant difference in CD86 intensity between the lesion and the distal tissue following cryogel-mediated LPS treatment (Fig. 3C–E). This indicates that the formation of an inflammatory lesion featuring CD86^+^ immune cells has occurred in the region adjacent to the cryogel scaffold. This inflammatory lesion was not observed in slices that were treated with control cryogels (Fig. 3D and E), indicating that the cryogel itself does not induce inflammation.

We also analysed the effect of cryogel-mediated LPS release on GFAP^+^ astrocytes, βIII-tubulin^+^ neurons and MBP^+^ myelin/oligodendrocytes (Fig. S3). LPS treatment had no effect on GFAP or βIII-tubulin intensity either from global medium-based treatment or cryogel-mediated treatment (Fig. S3D–E). However, LPS significantly reduced MBP intensity when delivered either via culture medium or via cryogel scaffold (Fig. S3F). This reduction in MBP intensity was only observed in the lesion site adjacent to the cryogel and not in the distal tissue, further supporting the formation of an inflammatory lesion which features demyelinated tissue. Demyelination did not occur in slices that were treated with control cryogels, again suggesting that the cryogel itself does not have a damaging effect on myelination.

### 3.4 Cryogel-mediated delivery of LPS can be used to screen immunomodulatory therapeutics *ex vivo*

**Fig. 4:**
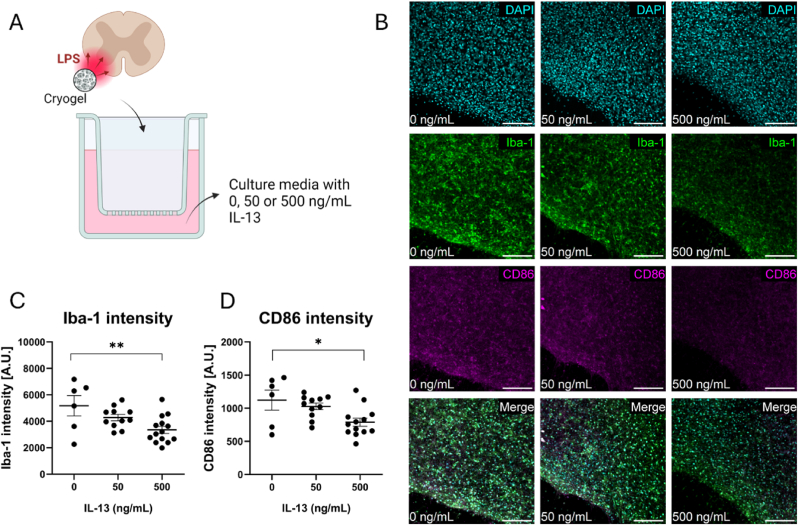
Cryogel-mediated delivery of LPS can be used to test immunomodulatory therapeutics. (A) Schematic depicting the experimental set-up whereby an inflammatory lesion is induced in organotypic spinal cord slices via cryogel-mediated delivery of LPS, and the immunomodulatory cytokine IL-13 is added to the media below the insert. (B) Representative photomicrographs of the inflammatory lesion in organotypic spinal cord slices treated with 0, 50 or 500 ng/mL IL-13 showing DAPI (cyan), Iba-1 (green) and CD86 (magenta). Scale bars represent 100 μm. Quantification of (C) Iba-1 and (D) CD86 intensity was performed using CellProfiler. Data represent mean ± SEM of n = 6-14 slices from 2 to 4 mouse pups per condition. Analysis by one-way ANOVA with Tukey's multiple comparison's test, ∗p < 0.05, ∗∗p < 0.01. (For interpretation of the references to colour in this figure legend, the reader is referred to the Web version of this article.)

IL-13 is a Th2 cytokine that is a known driver of an alternatively activated state in immune cells and is widely applied as an immunomodulatory therapeutic in preclinical studies of SCI [40,41], traumatic brain injury [42], MS [44] and stroke [45,46]. We confirmed the immunomodulatory action of IL-13 against LPS-induced inflammation in organotypic spinal cord slices by showing that IL-13 reduces Iba-1 and CD86 intensity in a dose-dependent manner (Fig. S4). We then examined the potential of our novel *ex vivo* model of neuroinflammation to act as a screening platform for immunomodulatory therapeutics by inducing an inflammatory lesion in organotypic spinal cord slices via cryogel-mediated LPS delivery and concurrently treating with IL-13 (Fig. 4A). Treatment with 500 ng/mL IL-13 significantly reduced Iba-1 and CD86 intensity in the lesion of cryogel-treated slices (Fig. 4B–D). Furthermore, IL-13 increased MBP and βIII-tubulin intensity in the lesion of cryogel-treated slices (Fig. S5). Taken together, these data indicate that this novel *ex vivo* model of neuroinflammation can act as an effective screening platform for immunomodulatory therapeutics (Fig. 4B–D).

## 4 Discussion

SCI is a devastating condition for which no curative therapy currently exists and more research into potential therapeutic strategies is urgently needed. The secondary inflammatory cascade is a major pathological event in SCI and immunomodulatory therapies show great promise for SCI repair. We have developed a novel *ex vivo* model of neuroinflammation, using cryogel-mediated delivery of LPS in mouse organotypic spinal cord slices, that is suitable for screening potential immunotherapeutics and holds potential for improving the efficiency of preclinical SCI studies.

Microglia are the resident immune cells of the CNS and are key orchestrators of the secondary inflammatory response after SCI [5,47]. Together with peripheral macrophages that infiltrate through the disrupted blood spinal cord barrier, these immune cells secrete proinflammatory cytokines and exacerbate recovery due to persistent inflammation for months after the primary injury. Microglia and macrophages exist on a dynamic activation spectrum ranging from an ‘M1-like’ neurodestructive state to an ‘M2-like’ neuroprotective state [[48], [49], [50]]. The ability to accurately model this complex multicellular inflammatory response is a crucial step towards developing novel immunomodulatory therapeutics that can drive M2-like activation. iPSC-derived microglia and macrophage cultures can recapitulate *in vivo* cellular properties, however they lack other CNS cell types which are essential in mimicking cellular interactions observed *in vivo* [5,14]. Neurospheroids are an improvement in this regard as they can be designed to contain multiple cell types [16,17]. For example, Cakir et al. designed human brain organoids that contained a vasculature network resembling the blood brain barrier [51]. The incorporation of functional microglia into CNS organoids is an ongoing challenge which limits their use for studying neuroinflammation. Although some groups have achieved this feat, the generation of microglia-containing organoids can take anywhere from 35 to 99 days and involves various costly growth factors and additives [52]. Furthermore, the use of terms such as ‘microglia-like cells’ highlights the uncertain biological relevance of these models [[53], [54], [55], [56]]. Compared to conventional *in vitro* models, organotypic slice cultures retain key structural and cellular features, including native tissue architecture, multicellular organisation, and cell–cell interactions, making them an improved platform for studying various aspects of the CNS microenvironment.

Critically, organotypic spinal cord slices contain a physiologically relevant number of endogenous microglia with anatomically relevant tissue microarchitecture [19]. Although this cannot fully replicate the complex *in vivo* SCI microenvironment, these features offer an improved platform for studying neuroinflammation *ex vivo* compared to conventional *in vitro* methods. The use of postnatal tissue for organotypic slice preparation is widely established due to the improved tissue viability and structural architecture compared to adult slice cultures [19,57]. Furthermore, functional microglia are established in the spinal cord by this time in development and thus postnatal slices offer an optimal platform for the study of neuroinflammation *ex vivo* [58]. We observed an increase in Iba-1 and CD86 intensity upon LPS stimulation of organotypic spinal cord slices, indicating that the slices contain functional microglia that are responsive to inflammatory stimuli (Fig. 1). We selected LPS as the inflammatory stimulus due to its robust and reproducible inflammatory effect, however various other factors including cytokines have been used to induce inflammation in organotypic slices. For example, Delbridge et al. treated organotypic brain slices with LPS, TNF-α and GM-CSF to demonstrate the molecular similarity of slice culture microglia to primary microglial cultures [24]. Giacco et al. reported that treatment of organotypic spinal cord slices with a cytokine cocktail of IL-1β, TNF-α, and GM-CSF triggered neuronal dysfunction via shortening of GABAergic synaptic currents, whereas treatment with LPS did not [26]. This may suggest that the inflammatory stimulus should be selected based on the desired outcome measure, as LPS may not be optimal in terms of studying synaptic dysfunction. Iba-1 and CD86 are physiologically relevant markers that allow for visual detection of proinflammatory microglia/macrophages [50,59,60]. Although broader analysis of secreted cytokines may be useful in some cases, direct immunostaining is preferred in the context of this study due to the ability to visualise the lesion site in the context of the surrounding tissue architecture, distinguish between inflamed and non-inflamed regions within the same slice, and confirm that the inflammatory response is indeed localised to the area adjacent to the cryogel.

Analysis of LPS release from the cryogel showed that LPS is rapidly released from the cryogel within the initial hours after loading (Fig. 2I). LPS carries a net negative charge due to the presence of phosphate and carboxyl groups, and thus LPS is not electrostatically bound to the inert PEGDA scaffold since there is no mechanism to retain it and control release [61,62]. Singh et al. reported that binding of LPS to the positively-charged KYE28 peptide decreased as the peptide was increasingly PEGylated, demonstrating that LPS and PEG do not interact [63]. We suggest a similar loading mechanism to that proposed by Eigel et al. whereby LPS is loaded into PEGDA cryogels by filling the macropores of the scaffold like a sponge, leaving it free for rapid release to the adjacent organotypic slice [29]. Given that LPS is not detected below the PTFE insert in the underlying culture medium (Fig. 2K), this suggests that LPS released from the cryogel is absorbed by the organotypic spinal cord slice when it is put in contact with the cryogel. Our calculations show that 0.049 ng LPS is directly delivered to the tissue via this method with the rapid release dynamics suggesting that this is delivered to the tissue within 4 h. The direct contact of the LPS-loaded cryogel with the organotypic slice is key to creating a focal inflammatory lesion that cannot be achieved through global LPS stimulation. More precise control over LPS loading and release may be achieved by modifying the PEGDA scaffolds with positively charged moieties [[64], [65], [66]]. However, the current methodology facilitated the generation of LPS-loaded cryogels that could successfully induce focal inflammation in organotypic spinal cord slices.

In our cryogel-treated slices, the observed increase in CD86 expression in Iba-1^+^ cells was limited to the region adjacent to the cryogel scaffold, indicating the formation of an inflammatory ‘lesion’ (Fig. 3). This is an important aspect of the SCI lesion to recapitulate since spared tissue surrounding the SCI lesion plays a key role in SCI repair [[67], [68], [69], [70]]. The clinical importance of tissue sparing was highlighted by Pfyffer et al. in a recent longitudinal cohort study whereby the extent of tissue sparing after SCI was correlated with functional improvement and thus could be used to guide clinical decision making moving forward [70]. Brennan et al. investigated the role of microglia in structural remodelling after high-thoracic (T3) SCI, and found that structural reorganisation in below-lesion circuitry involved synaptic pruning by microglia and drove autonomic dysfunction, highlighting that neuron-microglia interactions in spared lesion-remote tissue hold significant consequences for both SCI pathophysiology and SCI repair [71]. This therefore supports the use of our novel *ex vivo* model of neuroinflammation to study cellular injury mechanisms in a pathologically relevant microenvironment. Furthermore, our localised model may also facilitate a more nuanced investigation of drug effects on both inflamed and spared regions simultaneously which would be particularly valuable when screening for adverse drug effects on surrounding tissue.

Several limitations exist for this model that are important to discuss. Firstly, we did not observe any change in astrocyte reactivity (GFAP intensity) or neurodegeneration (βIII-tubulin intensity) following cryogel-mediated delivery of LPS (Fig. S3). Astrogliosis is a critical pathological event in SCI, as the formation of the glial scar around the lesion site forms a major barrier to regeneration [72,73]. Microglia-astrocyte crosstalk plays a key role in determining the phenotypic state of each cell under normal and pathological conditions, and thus an accurate representation of the reactive astrocytic state should be considered when modelling neuroinflammation [74]. LPS elicits highly variable responses on astrocyte reactivity depending on the concentration, exposure duration, and cellular context. This is likely due to the fact that astrocytes do not express TLR4 in appreciable levels [75]. In the literature, astrocytic responses to LPS are reported with exposure times ranging from as little as 5 min to 24 h, and concentrations from 10 ng/mL to 10 μg/mL [[75], [76], [77], [78]]. Although there are mixed reports on the ability of LPS to increase GFAP expression in astrocytes [[79], [80], [81], [82]], Norden et al. found that following peripheral LPS stimulation in mice, GFAP immunoreactivity in the hippocampus remained unchanged from 0 to 72 h despite the rapid increase in IL-1β, IL-6 and TNF-α expression in enriched astrocytes [83]. Furthermore, astrocytes can undergo functional reprogramming—such as changes in calcium signalling, glutamate uptake, or cytokine production—without robust changes in GFAP [84]. This suggests that astrocyte reactivity may indeed occur despite the unaltered GFAP immunoreactivity, and perhaps GFAP labelling alone is not sufficient to determine astrogliosis. Although additional molecular verification methods (e.g., qPCR or ELISA) may be more suitable for measuring cytokine-based astrocyte reactivity, measurement of secreted molecules from the media below the slices would not confirm the desired localised inflammatory effect in the same way as direct immunostaining. Costaining with complementary markers, such as S100β, could offer a broader view, although S100β has been shown to decrease following LPS stimulation *in vivo* and thus it may not be suitable for immunostaining purposes in this context [82].

Neurodegeneration is an additional pathological event in SCI that fuels the secondary inflammatory response through neuronal apoptosis and deposition of myelin debris [2]. Previous studies have shown that LPS reduces the number of neurons and reduces axon length in organotypic slice cultures [85,86]. However, this may be dependent on treatment duration [87], as we did not observe any effect of LPS on βIII-tubulin intensity, axon length or axon count in our model (Fig. S3). Furthermore, βIII-tubulin is a relatively stable cytoskeletal protein and may not reflect early signs of neuronal dysfunction, synaptic degradation, or dendritic pruning. This underscores the work by Giacco et al. and highlights that both inflammatory stimulus and detection method should be selected carefully based on study goal [26]. Importantly, we found that cryogel-mediated LPS delivery decreased MBP intensity in the lesion site (Fig. S3F). Demyelination occurs during the secondary injury phase of SCI and contributes to ongoing inflammation as myelin debris is cleared by activated microglia and infiltrating macrophages [2,4]. Plemel et al. showed that increased activation and proliferation of microglia following acute demyelination in the spinal cord restricted the spatial distribution of infiltrating macrophages, highlighting the interplay of demyelination and inflammation during the secondary injury cascade and the importance of recapitulating this process in an *ex vivo* model of neuroinflammation [88].

A further limitation of this model is the lack of functional vasculature and circulating immune components, which are key contributors to the inflammatory response in SCI. Although organotypic cultures do contain vascular networks [89], the inherent absence of dynamic circulation limits the ability to study certain aspects of immune trafficking and neurovascular interactions in this model. However, this model should be used in tandem with gold standard *in vivo* SCI models that more fully recapitulate these systemic features of SCI pathophysiology for more robust and efficient preclinical studies.

Finally, we applied IL-13 as a model therapeutic to showcase the ability of our model to screen potential immunomodulatory therapies. IL-13 binds a heterodimer receptor complex of IL13Rα1 and IL4Rα to initiate a JAK/STAT signalling cascade that drives alternative activation of immune cells following CNS trauma [90]. This ultimately leads to improved functional and histopathological outcomes including decreased astrogliosis, decreased demyelination and increased neuroprotection [[40], [41], [42],[44], [45], [46]]. We confirmed this immunomodulatory effect following both global LPS treatment (Fig. S4) and cryogel-mediated LPS treatment (Fig. 4) by showing that 500 ng/mL IL-13 counteracted LPS-induced increases in Iba-1 and CD86. Furthermore, 500 ng/mL IL-13 had beneficial effects on other CNS cells under inflammatory conditions as shown by increased MBP intensity and increased βIII-tubulin intensity following cryogel-mediated LPS treatment (Fig. S5). The advantage of confirming the therapeutic effect of IL-13 in the localised cryogel model over the global stimulation model is that global stimulation uniformly exposes all cell types throughout the organotypic slice to the inflammatory stimulus, eliminating regional specificity and making it difficult to study spatially restricted responses. Various studies have shown that the neuroprotective effect of IL-13 in the CNS is not due to direct action on neurons but rather due to the indirect action on activated microglia and macrophages [40,41,91]. One potential mechanism is the induction of apoptosis in activated microglia by IL-13, therefore reducing harmful contacts between activated microglia and vulnerable neurons [91,92]. Shin et al. showed that the expression of IL-13 b y activated microglia only occurred in the presence of neurons, and not with astrocytes or with microglia alone [91]. Therefore, the therapeutic action of IL-13 is dependent on microglia-neuron crosstalk and may not be fully realised unless investigated using a model where accurate cellular communication is faithfully recapitulated.

In conclusion, our study presents a novel *ex vivo* model of neuroinflammation that leverages cryogel-mediated delivery of LPS in organotypic spinal cord slices, providing an effective and efficient platform for screening potential immunotherapeutics for SCI. This model offers significant advantages over current *in vitro* approaches by preserving endogenous microglia and maintaining a physiologically relevant tissue microarchitecture, thus better capturing the complex cellular interactions essential for accurate modelling of SCI pathology. Our model effectively induces a localised inflammatory lesion surrounded by spared non-inflammatory tissue and demonstrates promising responses in terms of microglial activation and demyelination. The response to IL-13 demonstrates the potential utility for evaluating immunomodulatory therapies, which can drive neuroprotective and regenerative processes. Overall, this *ex vivo* system represents a valuable step forward in SCI research, enabling more realistic simulation of neuroinflammatory processes and supporting the advancement of targeted therapeutic interventions.

## CRediT authorship contribution statement

**Ciara M. Walsh:** Writing – original draft, Methodology, Investigation, Funding acquisition, Formal analysis, Data curation, Conceptualization. **Sophie Hill:** Methodology, Investigation, Formal analysis. **Ben Newland:** Writing – review & editing, Supervision, Resources, Methodology, Funding acquisition, Formal analysis, Conceptualization. **Dearbhaile Dooley:** Writing – review & editing, Supervision, Resources, Project administration, Methodology, Investigation, Funding acquisition, Formal analysis, Conceptualization.

## Declaration of competing interest

The authors declare no conflict of interest.

## Data Availability

Data will be made available on request.
