# Supplementary material for: Cryogel scaffolds for localised delivery of lipopolysaccharide in organotypic spinal cord slice cultures: A novel ex vivo model of neuroinflammation
Source: Mater Today Bio. 2025 Aug 19;34:102211. doi: 10.1016/j.mtbio.2025.102211 (PMC12396477; doi:10.1016/j.mtbio.2025.102211)
Supplement: Multimedia component 1 [file mmc1.docx]

**Supplementary material**

*
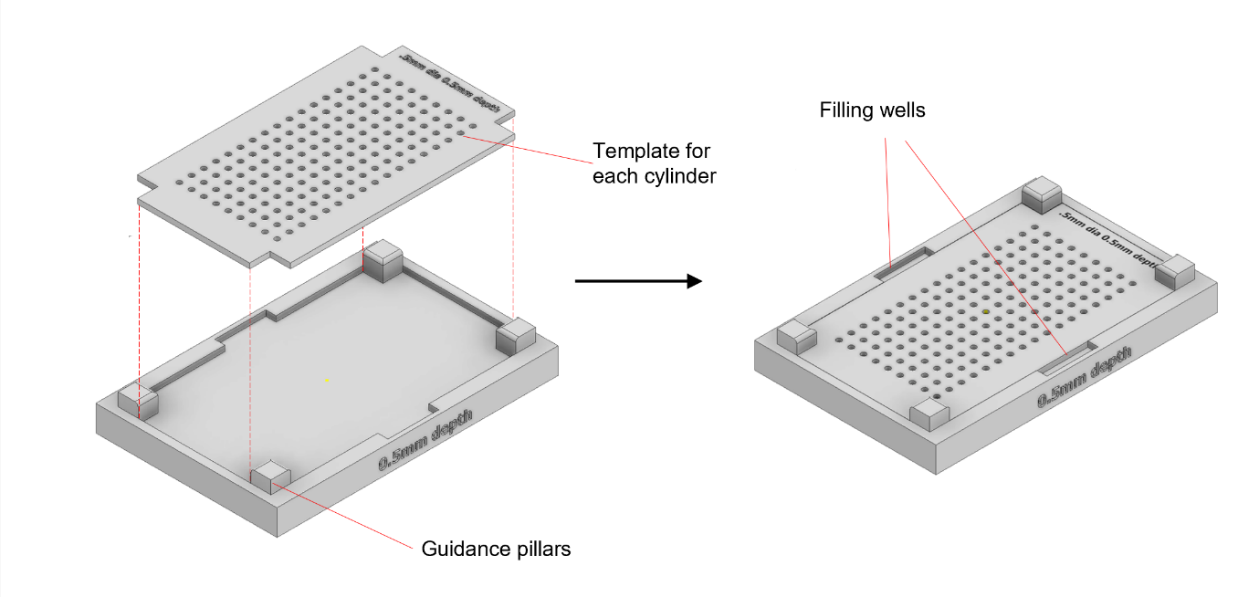
*

*Figure S1: Computer aided mould design. Images from computer aided design drawings of the two parts of the mould. This illustrates how they fit together to allow filling of multiple cylindrical moulds simultaneously.*


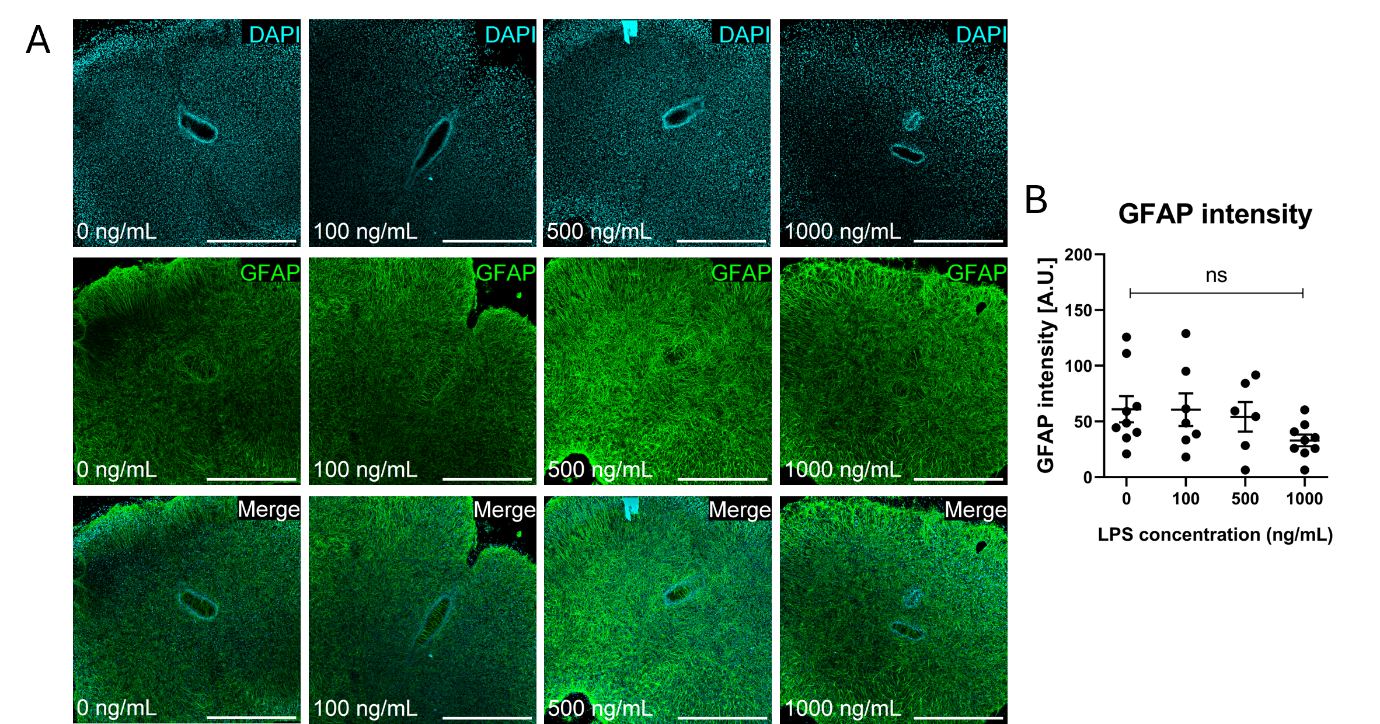


*Figure S2: LPS has no effect on GFAP expression in organotypic spinal cord slices after 24hr. (A)* *Representative photomicrographs of organotypic spinal cord slices showing DAPI (cyan) and GFAP (green) following treatment with 0 100, 500 or 1000 ng/mL LPS. (B) Quantification of GFAP intensity was performed using ImageJ. Scale bars represent 500 µm. Data represent mean ± SEM of n = 6-8 slices from 2-3 mouse pups per condition. Analysis by one-way ANOVA with Tukey’s multiple comparison’s test, ns p > 0.05.*


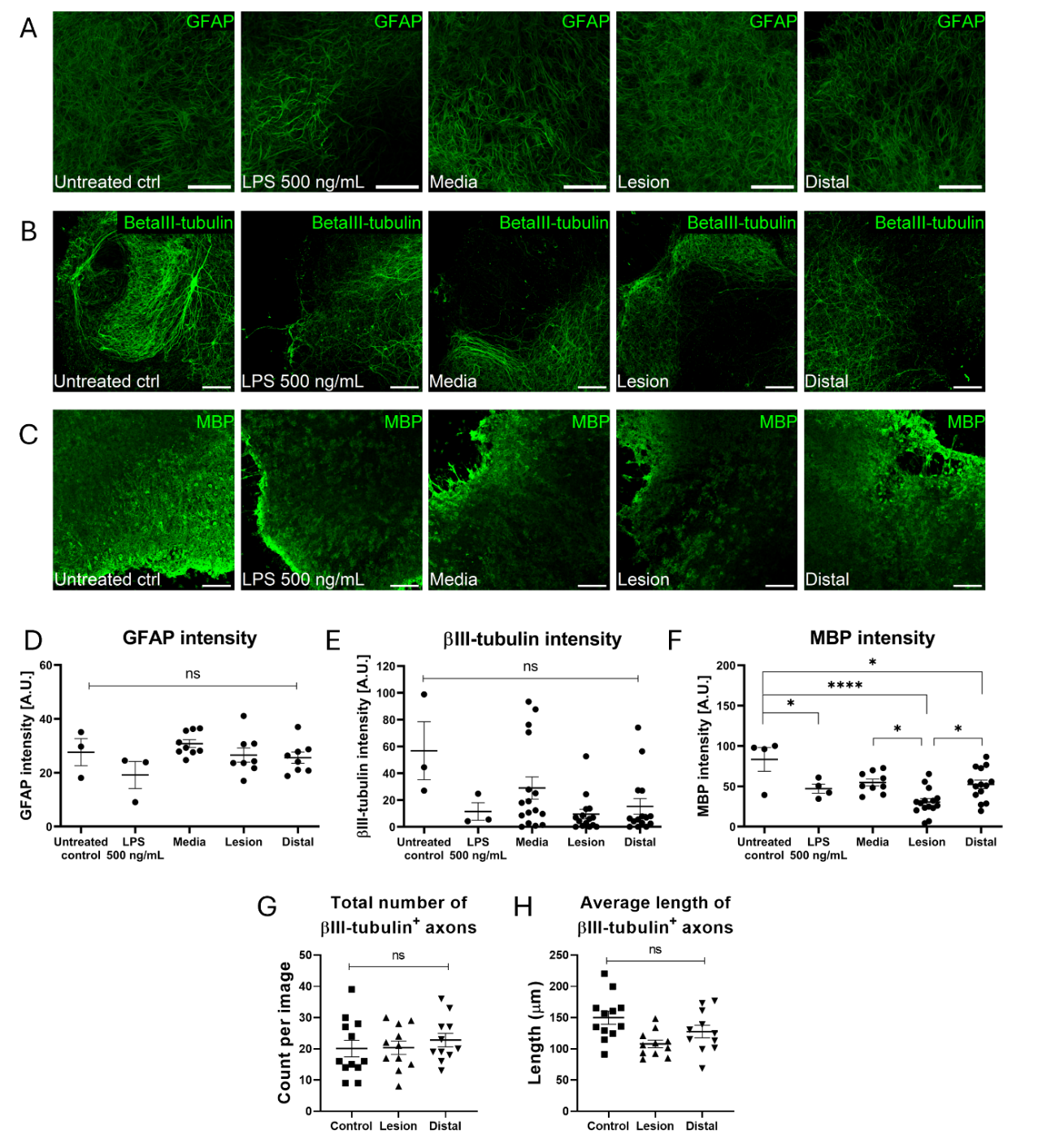


*Figure S3: Cryogel-mediated delivery of LPS reduces MBP expression in organotypic spinal cord slices. Representative photomicrographs of organotypic spinal cord slices showing (A) GFAP, (B) βIII-tubulin, or (C) MBP. Slices belong to the following treatment groups: Untreated controls, LPS 500 ng/mL (LPS added directly to media), Media (treatment with media-loaded cryogel), Lesion (lesion region following treatment with LPS-loaded cryogel), Distal (distal region following treatment with LPS-loaded cryogel). Quantification of (D) GFAP intensity, (E) βIII-tubulin intensity, (F) MBP intensity, (H) βIII-tubulin axon count, and (H) βIII-tubulin axon length was performed using ImageJ. Scale bars represent 100 µm. Data represent mean ± SEM of n = 3-15 slices from 2-4 mouse pups per condition. Analysis by one-way ANOVA with Tukey’s multiple comparison’s test, **** p < 0.0001, * p < 0.05, ns p > 0.05.*


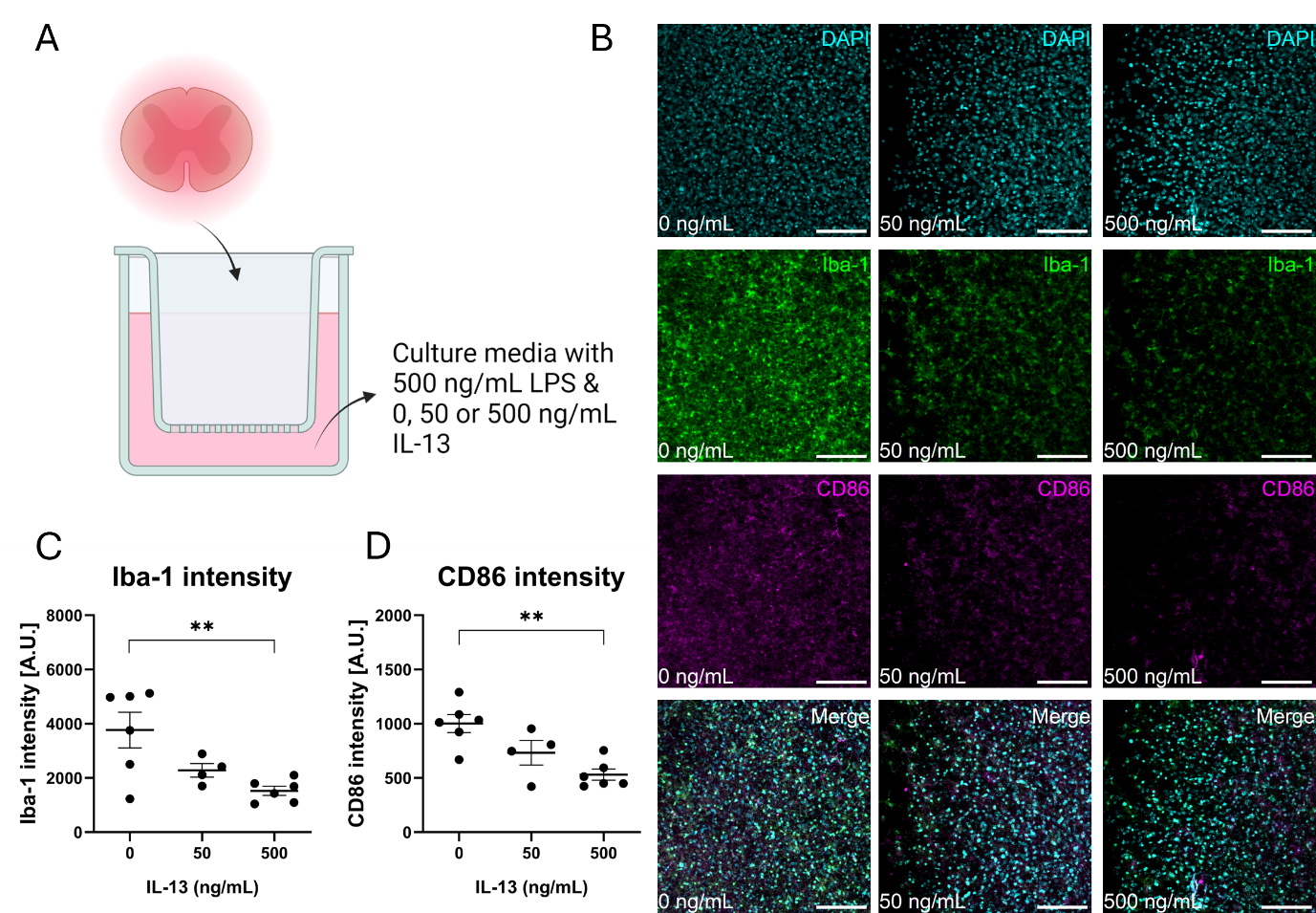


*Figure S4: IL-13 reduces LPS-induced inflammation in organotypic spinal cord slices. (A)* *Schematic depicting the experimental set-up whereby LPS and the immunomodulatory cytokine IL-13 are added to the media below the insert. (B) Representative photomicrographs of organotypic spinal cord slices treated with 0, 50 or 500 ng/mL IL-13 showing DAPI (cyan), Iba-1 (green) and CD86 (magenta). Scale bars represent 100 µm. Quantification of (C) Iba-1 and (D) CD86 intensity was performed using CellProfiler. Data represent mean ± SEM of n = 4-6 slices from 2 mouse pups per condition. Analysis by one-way ANOVA with Tukey’s multiple comparison’s test, ** p < 0.01.*
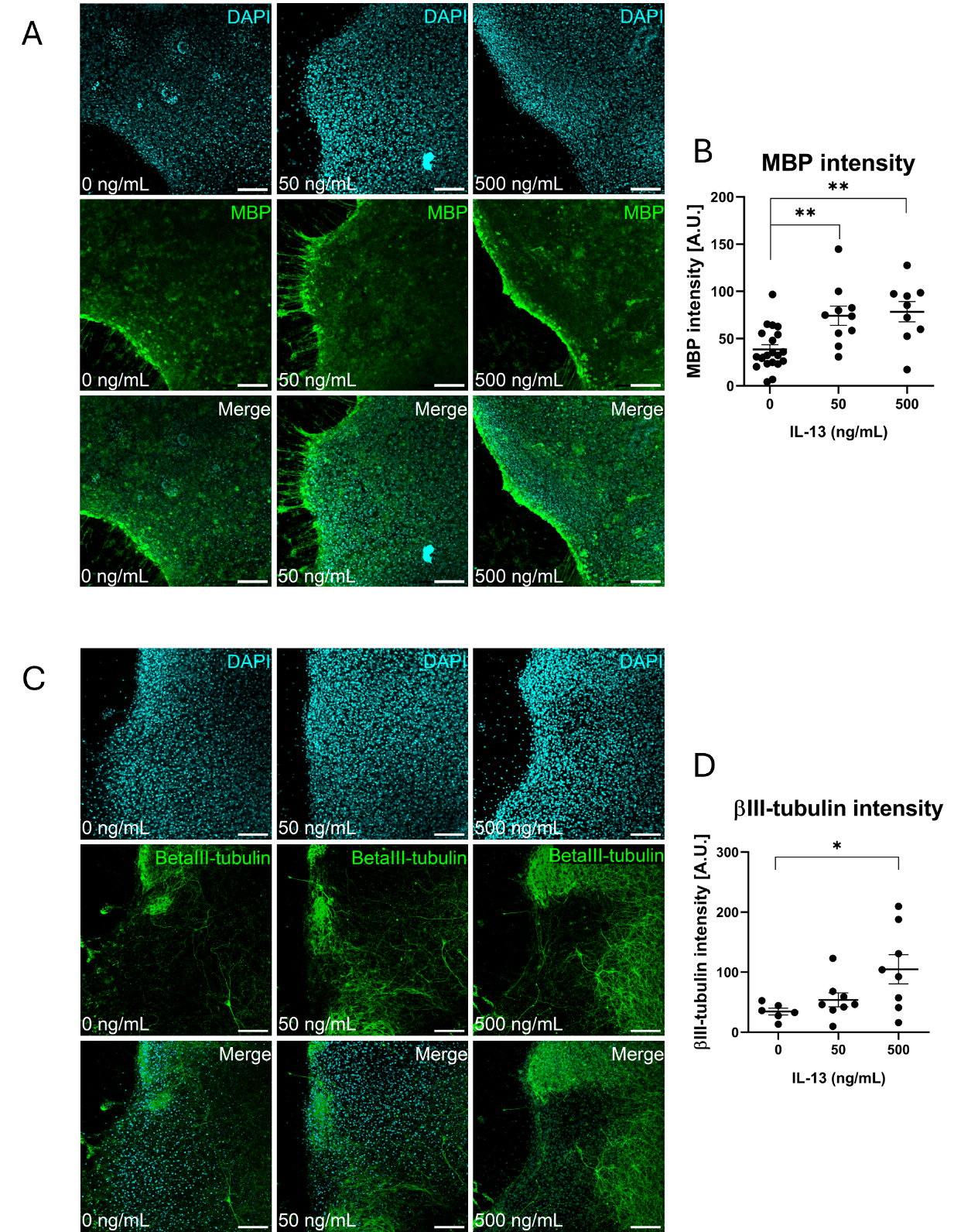


*Figure S5: IL-13 increases MBP and βIII-tubulin intensity following cryogel-mediated delivery of LPS. Representative photomicrographs of the inflammatory lesion in organotypic spinal cord slices treated with 0, 50 or 500 ng/mL IL-13 showing DAPI (cyan) and (A) MBP or (C) βIII-tubulin (green). Scale bars represent 100 µm. Quantification of (B) MBP and (D) βIII-tubulin intensity was performed using ImageJ. Data represent mean ± SEM of n = 6-14 slices from 3-4 mouse pups per condition. Analysis by one-way ANOVA with Tukey’s multiple comparison’s test, * p < 0.05, ** p < 0.01.*
